# Mitochondrial Dynamics: Working with the Cytoskeleton and Intracellular Organelles to Mediate Mechanotransduction

**DOI:** 10.14336/AD.2023.0201

**Published:** 2023-10-01

**Authors:** Danyuan Huang, Shuo Chen, Ding Xiong, Han Wang, Li Zhu, Yuanyuan Wei, Yuyu Li, Shujuan Zou

**Affiliations:** State Key Laboratory of Oral Diseases, National Clinical Research Center for Oral Diseases, West China Hospital of Stomatology, Sichuan University, Chengdu, China

**Keywords:** mitochondrial dynamics, cytoskeleton, mechanotransduction

## Abstract

Cells are constantly exposed to various mechanical environments; therefore, it is important that they are able to sense and adapt to changes. It is known that the cytoskeleton plays a critical role in mediating and generating extra- and intracellular forces and that mitochondrial dynamics are crucial for maintaining energy homeostasis. Nevertheless, the mechanisms by which cells integrate mechanosensing, mechanotransduction, and metabolic reprogramming remain poorly understood. In this review, we first discuss the interaction between mitochondrial dynamics and cytoskeletal components, followed by the annotation of membranous organelles intimately related to mitochondrial dynamic events. Finally, we discuss the evidence supporting the participation of mitochondria in mechanotransduction and corresponding alterations in cellular energy conditions. Notable advances in bioenergetics and biomechanics suggest that the mechanotransduction system composed of mitochondria, the cytoskeletal system, and membranous organelles is regulated through mitochondrial dynamics, which may be a promising target for further investigation and precision therapies.

## Introduction

Forces shape our universe as well as each organism; biological processes are always subject to and generate force [1, 2]. For instance, the extracellular matrix (ECM), such as that found in cartilage and bone, produces tension forces against cell-generated traction. The ECM increases in stiffness to generate corresponding resistance forces. When the general mechanical environment changes, the body responds with physiological or pathological changes. Stress fracture, the most common overuse injury in athletes, is a typical example of accumulated damage resulting from repeated submaximal force loading. Underloading also intervenes in the normal functioning of the body, such as seen in osteoporosis, owing to microgravity, disuse, and aging. Proper adjustments to the stress environment can stimulate tissues to grow in desired ways, which is the fundamental mechanism widely exploited in orthopedics and orthodontics [3, 4]. To perceive and respond to mechanical stimuli in nature, our body has evolved an exquisite neural system, well known as the reflex arc. However, little is known about how cells sense and react to mechanical stress.

Cells can sense mechanical inputs through mechanotransduction [5]. Transducing requires mechanosensing receptors, such as integrins or ion channels, to capture and translate mechanical stress into signaling events, eventually eliciting biological responses. These energy-consuming activities are driven by mitochondria. Mitochondria are cytoplasmic double-membraned organelles that consist of the outer mitochondrial membrane (OM), characterized as a smooth lipid bilayer, the inner mitochondrial membrane (IM), which folds inward to form the cristae, and the intermembrane space (IMS) between the OM and the IM. The OM allows metabolite exchange between the IMS and the cytosol, whereas the IM contains electron transport chain (ETC) proteins and encloses the mitochondrial matrix. The matrix is the place where metabolic processes, including the Krebs cycle, beta-oxidation, and reactive oxygen species (ROS) production take place [6, 7]. Mitochondria are involved in a variety of processes, including adenosine triphosphate (ATP) production, ROS production, apoptosis, autophagy, and intracellular calcium homeostasis [8-10].

Among all functions, supplying energy is regarded as the most important function of mitochondria. Approximately a century ago, Otto Warburg isolated and purified respiratory enzymes, and since then researchers have focused on understanding the ultrastructure of mitochondria and their redox components and metabolic pathways. Mitochondria were discovered by Albert von Kölliker in 1857 and named by Karl Benda in 1898. The term MITOCHONDRION is a splice of the Greek terms “*mitos*” and “*chondros*”, which mean “thread” and “granule”, respectively, indicating transformation of mitochondria. In living cells, mitochondria are highly dynamic and adapt morphologically to the diverse cellular processes mentioned above. Mitochondria can transform into tubular or interconnected networks by undergoing a series of complicated, but precisely controlled, membranous dynamic procedures termed mitochondrial fission and fusion.

In this review, we summarized the background information and current opinions on mitochondrial dynamics, and then illustrated the interactions between the dynamic regulation of mitochondria and the cytoskeletal system as well as membranous organelles. Finally, we annotated recent investigations of possible approaches to mitochondrial dynamics-mediated mechanotransduction. Mitochondrial dynamics could potentially serve as a drug target in diseases, especially those characterized as constantly experiencing variable mechanical environments, such as disuse and aging-related osteoporosis.

## Mitochondrial Dynamics: Fission, Fusion, and Beyond

Mitochondrial dynamics are not only the result of multiple stimuli like hypoxia and mechanical stress, but also the cause underlying normal cell functioning, such as cell deformation and mobility. Diverse mechanisms regulate mitochondrial dynamics; the consequences of which are mostly dependent on mitochondrial fusion and fission of either the OM or IM. Mitofusin 1 (MFN1) and MFN2 are the core regulators of OM fusion, whereas fission is regulated by dynamin-related protein 1 (DRP1). Several adaptors participate in OM fission by recruiting DRP1 to the OM, including mitochondrial fission 1 protein (FIS1), mitochondrial elongation factor 1/2 (MIEF1/2), and mitochondrial fission factor (MFF) [11].

Unlike the dynamic regulation of the OM, IM fusion and fission occur through a relatively subtle approach. IM fusion mainly involves two forms of optic atrophy 1 (OPA1) in mammalian cells. Specifically, OPA1 is proteolytically cleaved to aid fusion via two IM peptides, OMA1 and YME1L1, resulting in the long-transmembrane form (L-OPA1) and short-soluble form (S-OPA1) of OPA1 [12, 13]. The integral membrane protein L-OPA1 is believed to mediate IM fusion independently by anchoring to the mitochondria-specific lipid cardiolipin, whereas the stoichiometric level of S-OPA1 induces fast IM fusion [14]. Therefore, the loss of OPA1 often leads to fragmentation of the mitochondrial network [15]. However, there are contradictory results indicating that over-expression of S-OPA1 inhibits fusion activity and leads to mitochondrial fragmentation, but the underlying mechanism remains unclear [13, 16]. Additionally, another IM protein, mitochondrial protein 18 kDa (MTP18), can fragment mitochondria when its expression level increases, and the expression of MTP18 is interrelated with DRP1-induced mitochondria [17]. Consequently, depletion of MTP18 causes mitochondrial hyperfusion. MTP18 is distinct from the IM; it does not possess any IM motifs or domains. Hence, whether MTP18 acts as a ubiquitous IM fission mediator needs further research. In brief, mitochondrial membrane dynamics related to fission and fusion are crucial to mitochondrial homeostasis as well as the stability of the intercellular energy supply.

In addition to the regulatory proteins of mitochondrial fission-fusion, two stimuli widely utilized to modify the mitochondrial network morphology have drawn considerable attention, mitochondrial depolarization and increased intracellular calcium levels. These stimuli can induce small and rounded mitochondria, resulting in the speculation that both can induce mitochondrial fission. Nevertheless, Fung et al. [18] discovered that mitochondrial fragmentation resulting from increased calcium levels and depolarization was distinct. During calcium-induced mitochondrial fragmentation, inverted formin 2 (INF2) activity, which regulates actin assembly around mitochondria, leads to fission in an INF2-dependent manner. Mitochondrial depolarization, on the other hand, can initialize uncanonical mitochondrial fission in an INF2-independent pathway. Briefly, mitochondrial depolarization mediated by the carbonyl cyanide 3-chlorophenylhydrazone (CCCP) relies on the actin-related protein 2/3 (ARP2/3) complex to assemble transient actin clouds around depolarized mitochondria [18]. Thereafter, mitochondrial depolarization results in IM rounding and shape deformation. Previously, CCCP-induced mitochondrial deformation was characterized as a ring-like structure. A recent study using volume electron microscopy and live-cell imaging showed that the so-called ring-like structure was actually a three-dimensional disc with central invaginations, which formerly appeared as a “ring” in cross-sections [19]. Notably, compared with canonical mitochondrial fission and fusion, CCCP-induced morphological changes likely originated from the rounding of mitochondria [18, 19]. This alteration was DRP1-dependent, which was due to IM or cristae arrangement with the OM remaining intact [18]. In summary, CCCP-induced mitochondrial shape alteration might be a vital but independent switch between connected and fragmented mitochondrial networks. Treatment with other drugs that suppress mitochondrial function, such as oligomycin (an ATP synthesis inhibitor), can directly result in mitochondrial transformation [20]. Nonetheless, the relationship between mitochondrial morphology and function involves various factors, while the regulation of metabolic levels modifies mitochondrial dynamics through fission, fusion, morphological transformation, and beyond [21]. Mitochondrial dynamics do not always correlate with function. For instance, in *Drosophila* neurons, it is of vital importance for mitochondria to maintain normal function to sustain viability of the organism, while such processes are independent of mitochondrial inter-compartment distribution, suggesting that membranous dynamics and function may be separated in some cases [22].

**Figure 1.**
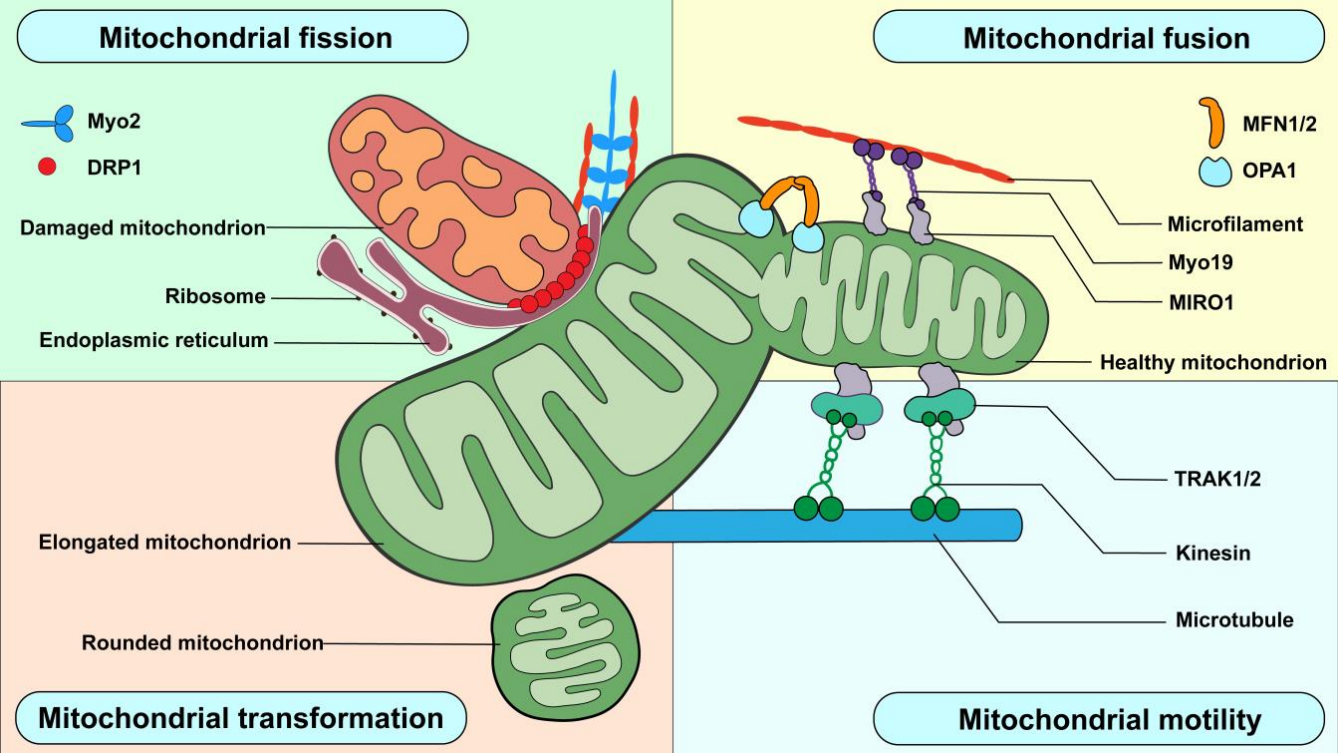
Four canonical forms of mitochondrial dynamics. Mitochondrial fission is mediated via DRP1 recruited at fission sites with the help of the endoplasmic reticulum and many cytoskeletal components. Eventually, the fission event results in two separate mitochondria. Mitochondrial fusion occurs when at least two mitochondria merge. This process requires MFN1/2 and OPA1 to fuse the OM and IM. Mitochondrial motility includes intracellular transport and positioning. These movements usually require the assistance of motor proteins, adaptors, and the cytoskeleton. Mitochondrial transformation refers to a series of morphological changes that are independent of mitochondrial fission or fusion, such as rounding and elongation of the mitochondria under certain conditions.

As mitochondria are recognized as the key component of intracellular calcium stores, multiple stimulations that elevate cytosolic calcium also cause changes in mitochondrial dynamics [23]. Despite the direct promotion of mitochondrial fission by facilitating classical DRP1 oligomerization and actin polymerization [24-26], increasing intracellular calcium levels also have considerable effects on mitochondrial dynamics through mitochondrial rho GTPase 1 (MIRO1). MIRO1, having two GTPase domains and two calcium-binding EF-hands, is a transmembrane protein located in the OM [27]. Calcium binds to a single EF-hand inducing mitochondrial shortening, a unique process that produces small and rounded mitochondria independently of DRP1, suggesting its distinctiveness compared to mitochondrial fission [27]. Therefore, calcium regulates mitochondrial morphology by facilitating fission and transformation (Fig. 1).

## Cytoskeletal system and mitochondria

Cells rely on two sets of networks to interact with the environment, the ECM, and the cytoskeletal system. The cytoskeletal system consists of the submembrane cell cortex, cytoplasmic skeleton, and nucleoskeleton, together with their interacting complexes. Although these networks exist within different cellular compartments, they share a close relationship in terms of both structure and function over space and time. The cell reacts to mechanical environment alteration through mitochondria-cytoskeleton interactions, involving mitochondrial dynamics, positioning, and function, regulated by the cytoskeletal network [28]. The cytoskeletal system is a dynamic, complex meshwork of interconnecting protein filaments that exists in all eukaryotes [29]. Strictly speaking, the cytoskeleton refers to the cytosolic network, which mainly consists of microfilaments (MFs, also known as F-actin), microtubules (MTs), intermediate filaments (IFs), and the new proposed component, septins.

F-actin is assembled from monomeric G-actin, mediated by actin nucleators and crosslinkers, such as formins, fascin, and ARP2/3 [30]. It can form highly ordered flexible structures that organize into linear bundles, two-dimensional (2D) networks, and three-dimensional (3D) gels [31]. F-actin is distributed throughout cells and forms cytoskeletal structures with diverse functions. Specifically, INF2-mediated nucleation of actin filaments promotes mitochondrial fission, whereas fascin-bound actin filaments shape into filopodia structures that cells use to probe the environment [32, 33]. The actin network shapes the morphology of the cell surface and maintains the mechanical stability of cells while participating in cytokinesis and assisting cell motility [34]. Myosins are actin-based motor proteins that are vital for muscle contractile function, and are powered by ATP hydrolysis to achieve actin-based movement [35]. F-actin also participates in the regulation of mitochondrial ROS production, dynamics, and positioning, and myosins have been implicated in fission events [36].

MTs typically begin to assemble from the MTs organizing center, and the minus ends of MTs are capped [37]. Cytoplasmic dynein (hereinafter referred to as dynein) and kinesin bind to the minus and plus ends, respectively, and function as directional motor proteins. These motors generate forces and move cargo along MT cytoskeletal tracks. MTs suppress the fission of mitochondria to which they are bound and provide mitochondria with transport and anchorage tracks [38, 39].

IFs are non-polar structures that increase the mechanical strength of cells and help in anchoring organelles [40]. These filaments, with an average diameter of 10 nm, are firmly bound and thus more stable than MFs. IFs, the support system or “scaffolding” for the cell and nucleus, functioning in the organization of intracellular 3D structures. Moreover, IFs also affect mitochondrial organization, morphology, lipid composition, and mitochondrial membrane potential (MMP) [41, 42]. MFs, MTs, and IFs are crosslinked by plectin [43]. Plectin regulates mitochondrial morphology and ROS production [44, 45].

**Figure 2.**
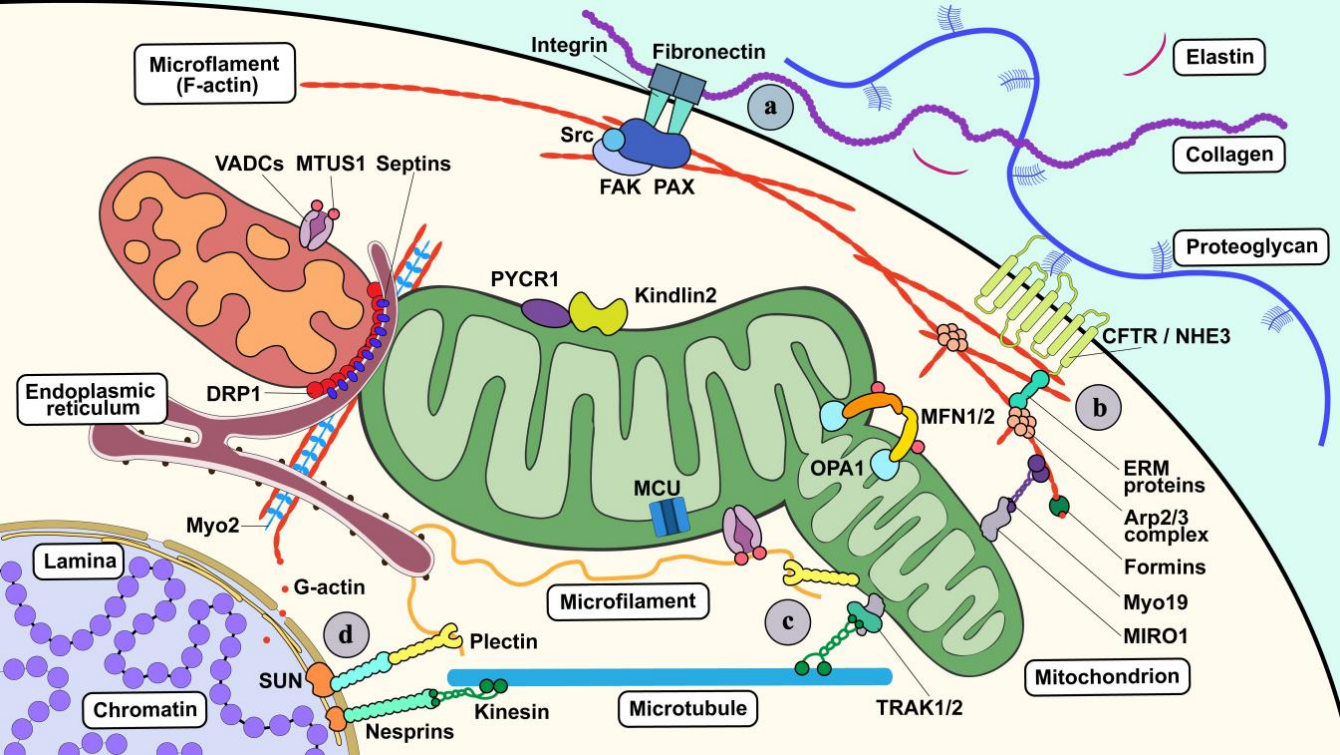
Interactions between mitochondrial dynamics and the cytoskeletal system. The ECM (a) and the cytoskeletal system consist of the cell cortex (b), cytoskeleton (c), and nucleoskeleton (d). Each component plays a role in the regulation of mitochondrial dynamics. (a) Fibronectin-bound integrins internalize mechanical signals from the ECM (especially fibrillar adhesion), regulating mitochondrial dynamics via the AMPK/mTOR pathway. PAX interacts with FAK and promotes FAK-Src binding, thereby enhancing the linkage of integrins to the actin cytoskeleton, which leads to alterations in mitochondrial dynamics. (b) Formins regulate actin polymerization in the cell cortex, whereas ARP2/3 complexes tend to form actin branching and capping. Both formins and ARP2/3 participate in the regulation of mitochondrial motility and positioning by enabling the cell cortex to be highly dynamic. (c) The cytoskeleton-mitochondria interplay is the core process that regulates mitochondrial dynamics. MFs (F-actin) form a network and scaffold, ensuring that all mitochondrial dynamic activities are under proper navigation and surveillance. MTs serve as the “highway” for mitochondria transport, and some associated proteins (e.g., MTUS1) take part in the modulation of mitochondrial fission and fusion via interaction with VDACs and mitofusins. IFs also form a fibrous skeletal network that provides mitochondria with anchoring and positioning sites; however, specific functions of IFs in the modulation of mitochondrial dynamics vary greatly due to their heterogeneity in different cell lineages. Plectins are a group of IF-associated proteins that link the mitochondria to the IF network, and their existence maintains mitochondrial fusion under supervision. In contrast, septins (e.g., Sept2) bind several constriction-related proteins to ensure normal mitochondrial fission. (d) Nucleoskeleton connects itself with the cytoskeleton through the LINC complex. Nesprin-1 and -2 are key components of the LINC complex, and they are also related to mitochondrial dynamics.

Septins are unique cytoskeletal components. They can be assembled into nonpolar filaments to form bundles, loops, and cage-like structures that contribute to the organization of intracellular compartments [46]. Septins also alter mitochondrial morphology [47]. These fibrous skeletal structures are able to rapidly grow or disassemble, depending on the physiological demands of the cell (Fig. 2).

### ECM - mitochondria interactions

The ECM is a 3D network composed of several types of macromolecules and minerals, including collagen, glycoproteins, and hydroxyapatite. The ECM provides structural and biochemical support to surrounding cells [48]. As multicellularity has evolved independently in different cell lineages, ECM components vary across different structures. Cell adhesion, intercellular communication, and regulation of cell differentiation are among functions of the ECM. Almost all types of living cells are exposed to various mechanical factors, such as pressure, tensile stress, shear stress, and stiffness of the surrounding ECM. These factors modulate cell function through the interplay between membrane receptors, including integrins at focal adhesions, and specialized binding sites on ECM fibers (such as Arg-Gly-Asp on type I collagen). Cells co-evolve with the ECM to rapidly respond to external stimuli and maintain homeostasis. For example, stem cells associated with the soft ECM show neurogenic abilities mimicking the brain; stem cells associated with harder ECM are myogenic mimicking muscle; and stem cells associated with stiff ECM initiate osteogenic differentiation simulating bone tissue [49].

The question of whether ECM mechanics influence mitochondrial function has been addressed, but there are limited data regarding whether ECM stiffness has an effect on mitochondrial structure and function. In cardiac myocytes, the baseline metabolic level is affected by ECM stiffness [50]. Moreover, fiber alignment and ECM stiffness regulate the adaptability of cells toward metabolic stress. In addition to mechanics, extracellular chemical conditions also influence mitochondrial behavior. For example, the mitochondria of tumor cells grown in Hanks’ balanced salt solution tend to exhibit a hyperfused status [51]. Mechanistically, protein kinase A phosphorylates DRP1 at Ser637, leading to DRP1 inactivation.

Furthermore, fibronectin-bound integrins enable specific internalization of fiber adhesion-mediated mechanical signals dependent on tensin. This internalization activity is characterized by mammalian target of rapamycin (mTOR) activation and lysosomal clustering. AMP-activated protein kinase (AMPK) deactivates tensin-dependent integrin in fibroblasts [52]. Interestingly, adhesion complexes have been found to participate in the regulation of mitochondrial function. The focal adhesion protein paxillin (PAX) interacts with focal adhesion kinase (FAK), which promotes FAK-steroid receptor coactivator (Src) binding and downstream pathways, linking integrins to the actin cytoskeleton [53]. PAX has also been shown to be mutated, amplified, or overexpressed in lung cancer cells, and the influence of the most common PAX mutations on mitochondrial dynamics has been studied. Specifically, A127T, P233L, and P487L PAX mutations were found to impact focal adhesions, whereas P233L and D399N mutants were associated with B-cell lymphoma 2 localized to the mitochondria with DRP1 and MFN2. Notably, A127T and P487L mutants were found to trigger hyperfused mitochondria with higher density, whereas the D399N mutant showed similar phenotypes of mitochondrial fragmentation with lower density [54]. The effects of these mutations were tested *in vitro* using HEK-239 cells derived from human embryonic kidney epithelium. It is still unclear how these PAX mutations induce changes in mitochondrial dynamics, which further affect mitochondrial function and energy production [55]. Inhibition of phosphoinositide 3-kinase promotes the recruitment of phosphorylated FAK around mitochondria, thereby increasing the turnover and assembly of focal adhesions. This pathway is regulated by serine/threonine kinase (Akt), mTOR, and oxidative phosphorylation [56]. However, investigation is required to clarify the detailed molecular mechanisms involved. In addition, it is not known whether these findings are applicable to other molecular pathological subtypes of lung cancers and even to other cancer cell lines with PAX mutations (such as acute lymphoblastic leukemia), which may have potential research value.

### Cell cortex and mitochondria

The cell cortex (actomyosin cortex) is a special protein layer located in the plasma membrane. It works as a regulator of surface properties and membrane behavior [57]. In particular, the cell cortex generates tensile stress beneath the plasma membrane, which is a crucial structural basis for cell morphological alterations and locomotion. In mammalian cells, the cortex is an actin-rich network composed of F-actin, myosin, and actin-binding proteins [58]. The actomyosin cortex is attached to the plasma membrane through the membrane-anchoring protein ezrin-radixin-moesin (ERM), which is pivotal to the control of cell morphology [59]. The cell cortex can rapidly modify its proteins, which provides strong mechanical rigidity and plasticity, essential properties that are closely related to the function of the cell cortex.

The cortex meshwork is dynamically regulated by many proteins, including formin, which aids in actin polymerization, ARP2/3 complexes, which initiate actin branching allowing the actomyosin cortex to form a fractal structure, and various capping proteins [60]. This diversity of components contributes to the uniqueness of the actomyosin cortex in certain specialized cells. For instance, the cortex of red blood cells consists of a 2D cross-linked elastic network formed primarily by F-actin, ankyrin, and spectrin [61]. In neuronal axons, the cell cortex appears as an array of periodic rings formed by F-actin and spectrin, whereas the cortex forms a helical structure in the sperm flagellum [62, 63]. In yeast, nuclear migration protein 1 plays a specific role in anchoring mitochondria to the actomyosin cortex when mitochondria are preferentially distributed during cell division [64]. However, researchers are far from determining how the interactions between mitochondria and the cell cortex affect mechanotransduction.

### Cytoskeleton and mitochondrial dynamics

F-actin modulates mitochondrial fission by enhancing the activity of DRP1 [65]. DRP1 oligomers form a circular structure around the pre-division site of the OM, initiating fission events, inducing mitochondrial contraction, and eventually leading to division [66]. However, the mitochondrial circumference is generally wider than that of the DRP1 loop. Therefore, F-actin is required to pre-constrict mitochondria at mitochondria-endoplasmic reticulum (ER) contact sites (MERCs). This narrows the cross-sectional diameter of the mitochondria such that DRP1 can begin mitochondrial fission [67].*In vitro* studies have shown that F-actin alone can promote DRP1 activity, possibly because F-actin contributes to the polymerization of DRP1 oligomers and maturation of the rings [68]. Thus, a knockdown of the actin depolymerization protein cofilin1 results in accumulation of DRP1 and mitochondrial fission increases [69]. Nevertheless, another study reported that while downregulation of cofilin enhanced the accumulation of DRP1 in the mitochondria, it also caused mitochondrial hyperfusion [70]. This suggests that the DRP1-independent pathway regulates mitochondrial fission.

Mitochondrial morphology and dynamics are also controlled by the actin-nucleating protein INF2. Depletion of INF2 causes the loss of F-actin, leading to an increase in mitochondrial length [71]. Another nucleator, Spire1C, along with INF2, can form short actin filaments localized to MERCs [72]. These short filaments generate a force, tightening the ER tubules around the mitochondria [73]. The actin-based motor non-muscle myosin II (NMMII) might be responsible for the tightening as it is localized to the MERCs, and knockout of NMMII leads to elongated mitochondria [74]. Additionally, non-muscle myosin isoform IIC (NMIIC) was recently recognized as a fission-associated motor protein [75]. Mutations in the gene encoding NMIIC reduce mitochondrial fission [75]. NMIIC mutations are associated with peripheral neuropathy and hearing loss, indicating that defects in mitochondrial dynamics may play a role in these diseases [75].

With the assistance of the actin branching protein ARP2/3, F-actin can also be transiently assembled on hyperfused regions of the OM to modulate stress-induced mitochondrial fission [76]. Actin assembly facilitates DRP1 recruitment, followed by rapid fission within minutes [77]. This function of ARP2/3 and F-actin may aid the rapid increase in mitochondrial fission in mitotic prophase [76].

In eukaryotic cells, the structure and function of MTs and mitochondria are closely related [39]. Transmission electron microscopy observations showed that alpha- and beta-tubulin localize to the OM, and immunoprecipitation results confirmed that they are associated with voltage-dependent anion channels (VDACs) in the OM [78]. Interference with MT assembly reduces mitochondrial motility, hence affecting mitochondrial fission and fusion [77]. In *Dictyostelium discoideum* cells, treatment with nocodazole resulted in MT depolymerization and reduced mitochondrial fission and fusion events thereafter [79]. In *Schizosaccharomyces pombe*, the depolymerization or destabilization of MTs increased the mitochondrial fission rate [80]. In contrast, lowering the frequency of MT catastrophe reduced the mitochondrial fission rate, leading to elongated MTs and hyperfused mitochondrial phenotypes in *Schizosaccharomyces pombe* [39].

MT-associated proteins also regulate mitochondrial morphology and dynamics. For instance, depletion of mitochondrial tumor suppressor 1 (MTSG1, also MTUS1), which is usually localized to the OM via MFN1/2, resulted in shorter and rounder mitochondria in endothelial cells, suggesting its role in maintaining the balance between fission and fusion events [81]. In *Saccharomyces cerevisiae*, the removal of Mmb1p, another MT-mitochondria linker protein, caused unopposed mitochondrial fission [82]. These examples indicate that the connection between mitochondria and MTs is pivotal for dynamin-1 (yeast DRP1)-mediated mitochondrial fission [39].

IFs vimentin, desmin, and the linker protein plectin are associated with mitochondria [83, 84]. Vimentin can directly bind to mitochondria [73]. Vimentin depletion leads to swelling and fragmentation of mitochondria and significantly reduces the level of α-tubulin attached to mitochondria, indicating that vimentin may also regulate the interaction between mitochondria and MTs [84]. Desmin exists on mitochondria-associated membranes and interacts with several mitochondrial proteins [85]. Knockout of the desmin-encoding gene *DES* causes mitochondrial clustering and disorganization [85, 86], while depletion or mutation of desmin and its binding proteins plectin and myotubularin leads to abnormal mitochondrial organization [42, 83, 87, 88]. Keratins (especially keratin-18) are IFs that promote proper organization of mitochondria [89, 90]. In skeletal myoblasts, an isoform of plectin, P1b, associates IFs with the mitochondria [91]. Knockdown of P1b causes upregulation of MFN2, consequently leading to an increase in the width of mitochondrial Z-disk wrapping [91]. However, whether P1b is directly tethered to MFN2 and how P1b alters mitochondrial dynamics requires further investigation. In neurons, mitochondrial organization relies on interactions with vimentin and other associated proteins. Vimentin phosphorylation modulates the anchoring of mitochondria to IFs [92]. Vimentin expression, controlled by micro-RNA-124, also regulates mitochondrial locomotion [92]. Complete loss of IF-neurofilament light increases the motility of neuronal mitochondria [93]. Conversely, disease-linked aggregation of IFs and associated proteins usually causes a marked reduction in mitochondrial motility [94]. In conclusion, IFs regulate intracellular mitochondrial organization by anchoring mitochondria through direct or indirect interactions or through IF-associated proteins to modulate mitochondrial dynamics.

Septins, another cytoskeleton-associated protein similar to plectins, also cause changes in mitochondrial morphology [95]. Septin 2 (Sept2) directly binds to DRP1 and localizes to mitochondrial contraction sites. In mammalian cells, Sept2 depletion reduces the localization of DRP1 to the OM, leading to hyperfused phenotypes [96]. Silencing the gene encoding Sept7 also causes hyperfusion of mitochondria, probably resulting from the accidental co-depletion of Sept2 [95, 96]. However, the mechanism by which septins enhance DRP1 recruitment to fission sites remains unknown.

Recently, kindlins have been shown to participate in mechanotransduction between the ECM and mitochondria. Kindlins are FERM domain-containing proteins consisting of three members (kindlin 1-3), all of which are capable of binding to the membrane distal XXY motif of integrins [97]. Kindlin1 and 3 are expressed in the epithelium and hematopoietic system, respectively, whereas kindlin2 is widely distributed outside the hematopoietic system [98]. Kindlins work as an interaction bay for integrin-linked kinases, such as LIM and senescent cell antigen-like-containing domain protein 1 (LIMS1), PAX, the Parvin complex, and the ARP2/3 complex [98]. These proteins are recruited to kindlins and thereby bind to integrins as a complex. Kindlin2 can directly bind to F-actin, but there is no evidence of intramolecular tension across kindlins [99]. One possibility is that kindlins associate with integrins through actomyosin to internalize the extracellular mechanical milieu [100]. In other words, kindlins are likely to stabilize cell adhesion by regulating actin dynamics.

Furthermore, Guo et al. [101] reported that kindlin2 localizes not only at focal adhesions, but also in mitochondria. Mitochondrial kindlin2 interacts with pyrroline-5-carboxylate reductase 1 (PYCR1), a mitochondria-anchored enzyme crucial for proline synthesis [101]. Proline synthesis is a classic metabolic axis that is closely related to mechanotransduction from the ECM. On stiffer ECM, kindlin2 enhances the structural stability and activity of PYCR1, thereby increasing PYCR1 levels and proline synthesis [102]. This mechanism explains the high level of PYCR1 in advanced lung adenocarcinomas characterized by a stiffer tumor stroma [102]. In addition, proline plays a significant role in collagen synthesis, which indicates a potential interrelationship between mitochondrial kindlin2 and collagen. In summary, mitochondrial kindlin2 shows a feedforward mechanism in which stiff ECM may stimulate cells to promote ECM deposition and remodeling to further stiffen the ECM. This mechanism may provide a novel understanding of bone metabolic diseases and cancer metastasis.

### Nucleoskeleton and mitochondrial dynamics

Recently, the cytoskeleton has been accepted as a key modulator of nuclear functions. It is now recognized that nuclear actin participates in chromatin modification and transcriptional regulation with the assistance of several myosins, nuclear factors, and actin polymerization regulators. Generally, more than 30 proteins that affect actin polymerization have been found in the nucleus [103], including proteins previously considered components of the nucleoskeleton [104]. These findings redefine the concept of the nucleoskeleton from a rigid nuclear scaffold to an interconnected dynamic network. This network contains actin and actin-bound machinery, including chromatin, nucleic acids, and various protein complexes. In fact, it is no surprise that cytoskeletal proteins participate in the regulation of the mammalian genome because actin-like proteins of prokaryotes are widely involved in chromatin segregation [105]. This is also the case in mitochondria; for example, beta-actin regulates the separation of mitochondrial DNA (mtDNA) [106].

**Figure 3.**
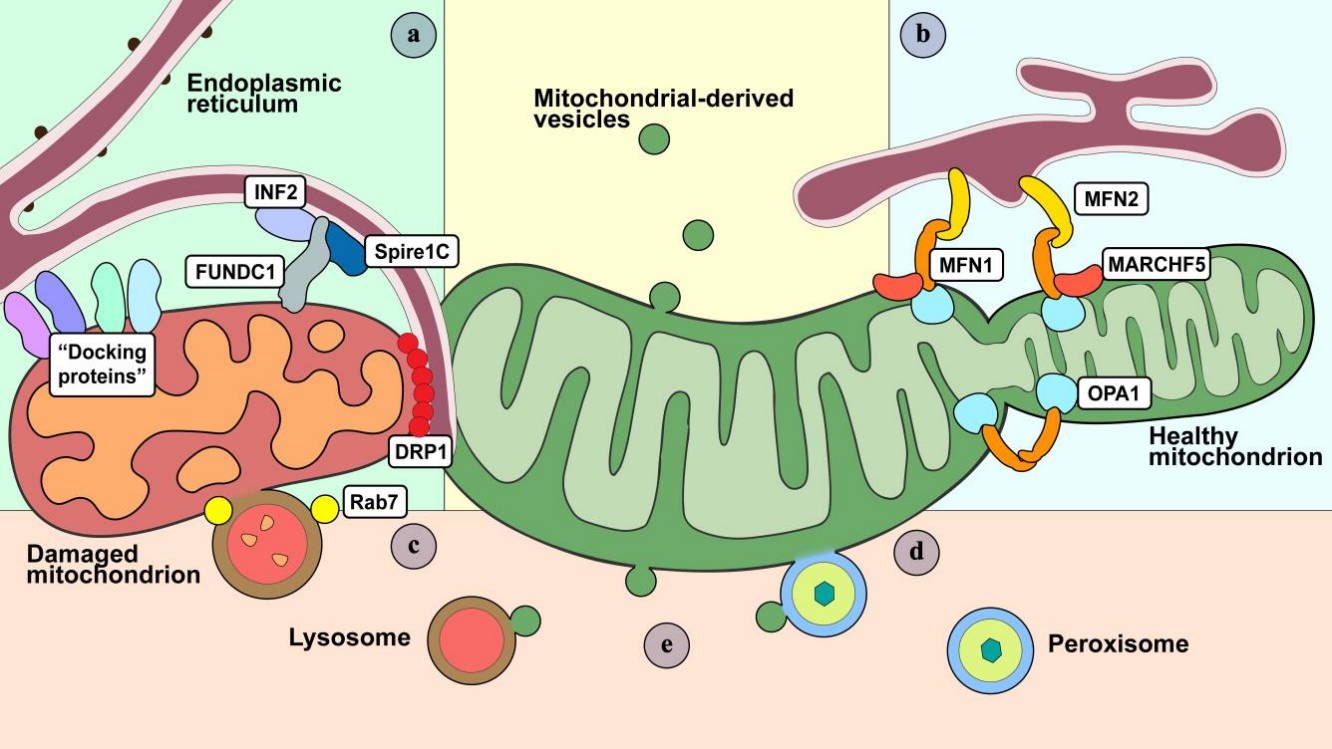
Membranous organelles participate in the dynamic regulation of mitochondria. (a) MAMs mark, initialize, and facilitate mitochondrial fission and fusion. For mitochondrial fission, a protrusion from the ER membranes, termed the ER tubule, is tethered to the fission site, followed by formation of F-actin scaffolds induced via INF2 and Spire1C. Thereafter, DRP1 proteins are recruited to the constriction ring with the assistance of “docking proteins” (such as Fis1, Mff, and MiD49/51) and cytoskeletal components. FUNDC1 also serves as a docking-like protein that mediates mitochondrial fragmentation via localizing the OM to the ER. (b) For mitochondrial fusion, with the induction of MARCH5, ER can be tethered to mitochondria via ER-MFN2 connected to mitochondrial MFN1/2, establishing a mitochondria-ER contact site to sustain further OM fusion. OPA1 initiates IM fusion in the presence of MFN1 instead of MFN2. (c) Rab7 is the most important GTPase protein that regulates mitochondria-lysosome interactions. Rab7 is likely to modulate mitochondrial fission events in a DRP1-dependent manner. (d) Peroxisomes are observed to intimately interact with the OM, and deformation of mitochondrial morphologies presents in several congenital peroxisomal diseases, indicating peroxisomes are potential regulators of mitochondrial dynamics. (e) MDVs shuttles cargo to lysosomes and peroxisomes as a means of mitochondrial quality control.

Notably, there exists a multi-subunit protein complex termed the linker of nucleoskeleton and cytoskeleton (LINC) complex comprising KASH and SUN domain proteins that spans the nuclear envelope and links the nuclear lamina to the cytoplasmic MT, actin, and IF networks [107]. This complicated structure is morphologically and functionally related to the mitochondria. Lindenboim et al. [108] illustrated that nesprins-2, a key component of the LINC complex serve as mediators regulating perinuclear mitochondrial positioning and mitochondrion-initiated apoptosis under mechanical stress. Their findings are supported by the *in vitro* study of Hao et al. [109], who reported that removal of nesprin-1/-2 impairs the positioning and dynamics of endomembranous organelles such as mitochondria. Nevertheless, *in vivo* studies are still needed to validate the mechanism by which nesprins regulate mitochondrial dynamics as well as to uncover their role during the development of diseases.

## Mitochondria and Membranous Organelles

In most cases, cells possess a set of exquisite mechano-transducive systems to accomplish cellular mechano-sensing. This system consists of not only the cytoskeletal system but also multiple endomembranous organelles. On one hand, compartmentation and specialization of organelles are beneficial for the structural and functional organization of cells, on the other hand, specialized organelle functions must be coordinated and integrated for cell physiology. To date, various mechanisms focusing on organellar interactions have been revealed, such as vesicle traffic-mediated interactions between heterotypic organelles. Moreover, membrane contact is a key interaction mechanism. Interorganelle membrane contact sites are contacts formed between the membranes of two distinct organelles in close proximity, allowing for intracellular communication. Membrane-membrane contact sites have been detected in almost all types of organelles that can mediate multiple physiological functions, including metabolite trafficking and signal transduction. As the main generator of all cellular activities with high dynamics, it is not surprising that mitochondria are pivotal in endomembranous contact events (Fig. 3).

### Mitochondria-associated membranes regulate mitochondrial dynamics

Mitochondria-associated membranes (MAMs) are the part of the ER that is reversibly tethered to mitochondria. MAMs play a role in the regulation of material exchange between the ER and mitochondria, together with the modulation of calcium homeostasis and mitochondrial function. Thus, aberrant MAMs are associated with neurodegenerative and metabolic disorders [110]. In recent years, the relationship between MAMs and mitochondrial dynamics has been extensively studied. It is already known that mitochondrial fusion is regulated through MFN1 and MFN2, which interact between mitochondria via their HR2 domains to tether two opposing mitochondria [111]. MFN1/2 can form oligomers or hetero-oligomers to initiate mitochondrial fusion [112]. MFN1 localizes only to the mitochondria, while MFN2 is present in both MAMs and mitochondria [113]. Whether the ER mediates mitochondrial fusion or directly forms MAMs with mitochondria likely depends on localized MFNs. The E3 ubiquitin-protein ligase MARCH5 (also known as the mitochondrial ligase MITOL) modulates MAM formation and ubiquitinates mitochondria-MFN2 instead of ER-MFN2. Takeda et al. [114] found that MFN2 K192 is the major ubiquitination site of MARCH5. However, OPA1-dependent IM fusion relies on MFN1 rather than MFN2 [115]. This indicates a possible communication between the two organelles during mitochondrial fusion. Meanwhile, the interaction of MFN1 with OPA1 seems likely, considering the recently confirmed MFN topology [116].

Here, MARCH5 was annotated as playing a controversial role in the regulation of mitochondrial dynamics. MARCH5 regulates mitochondrial morphology through polyubiquitination of DRP1 [117]; however, inhibition of MARCH5 can lead to mitochondrial fusion [118]. A possible explanation for this contradiction is that MARCH5-dependent modulation of DRP1 might vary with cellular conditions, such as oxidative stress or glucose status. Compared to mitochondrial fusion, recent studies have focused more on the interrelationship between MAMs and mitochondrial fission mediated by DRP1 [119]. Dynamin2 cooperates with DRP1 to constrict and separate mitochondria [120]. At MERCs, DRP1 forms oligomer docking to mitochondrial dynamic proteins (such as FIS1, MFF, and MIEF1/2) localized in MAMs before fission events [121]. Nevertheless, there are different opinions on the role of MIEF1/2 in mitochondrial dynamics [122, 123]. Recently, Lin et al. [124] found that the FUN14 domain-containing 1 protein (FUNDC1) on the OM could localize at MERCs and interact with the ER protein calnexin under hypoxic stress to start the accumulation of FUNDC1. Wang et al. [125] showed that FUNDC1 is required for mitochondrial fission. Specifically, FUNDC1 recruits DRP1, initiating mitochondrial fission in response to hypoxic stress. In addition, both ER-bound INF2 and mitochondria-anchored Spire1C localize to MERCs to modulate actin assembly necessary for DRP1 recruitment and oligomerization [121, 126, 127]. The interactions of these “docking proteins” with the cytoskeleton and MERCs have been clearly recognized as the basic regulators of mitochondrial fission. Further investigations are needed to reveal additional potential regulators and their interlinkages with fission machinery.

Studies focusing on how the ER recognizes and labels the mitochondrial fission site have provided breakthroughs. In mammalian cells, Mitochondrial nucleoids have been identified in MERCs [128]. Recently, live cell imaging and high-resolution microscopy have demonstrated that mtDNA replication is associated with MERCs, indicating that forming MERCs might guide the precise distribution of duplicated mtDNA into separated daughter mitochondria [129]. These novel findings suggest that mtDNA replication may occur one step earlier than the formation of MERCs during mitochondrial fission. More solid evidence is required to support this result. If the evidence is conclusive, how the signals of mtDNA replication are transmitted from the mitochondrial matrix to the MAMs, triggering MERC formation, is the next issue worthy of attention.

### Dynamic interactions between lysosomes and mitochondria

The results of 2D and 3D electron microscopy and correlative light electron microscopy (CLEM) have proven the existence of mitochondria-lysosome contact sites (MLCs) within various cell lines under healthy conditions [130, 131]. Moreover, CLEM combined with focused ion beam scanning electron microscopy showed stable contact between Lamp1-/dextran-positive vesicles and mitochondria [76, 132]. These contacts were reported to regulate the transfer of lipids, calcium, and iron, along with the modulation of mitochondrial fission [133]. Additional studies are required to elucidate the mechanisms by which MLCs regulate these cellular events.

Tethering between mitochondria and lysosomes is regulated by a variety of proteins on both membranes. The small GTPase Rab7 protein is a major switch in lysosomal dynamics. Inactive Rab7 is bound to GDP and located in the cytoplasm; once activated, it is transferred to lysosomes and binds to GTP. Constant switching between the two states allows Rab7 to regulate the tethering and release of MLCs. Generally, MLCs regulate mitochondrial fission, which is mostly characterized by Lamp1-positive vesicles instead of early endosomes and peroxisomes [131]. Inhibition of Rab7-GTP hydrolysis utilizing GTP-bound mutants, such as Rab7-Q67L or TBC1D15, disrupts the formation of MLCs, decreases the mitochondrial fission rate, and segregates the mitochondrial network [131]. Interestingly, lysosome-related mitochondrial fission is regulated by DRP1. Recently, a brain-enriched DRP1 isoform consisting of four alternative exons, DRP1ABCD, was discovered to be associated with Lamp1-positive vesicles and localized to MLCs [134]. The accurate localization of DRP1ABCD relies on acidification rather than the proteolytic activity of late endosomes or lysosomes, suggesting that DRP1 isoforms might have played unknown roles in the establishment of MLCs [134]. Therefore, MLCs modulate mitochondrial dynamics by marking fission sites and regulating the fission rate.

MLCs represent a novel mechanism of mitochondrial quality control (MQC) [82, 135]. In this case, MLCs not only refer to direct membrane contacts between lysosomes and mitochondria, but also include signaling crosstalk between these two organelles and indirect membrane contacts via release of lysosome-targeted mitochondrial derived vesicles (MDVs). Soubannier et al. first reported that MDVs shuttled mitochondrial cargos to lysosomes as an early response to oxidative stress [136]. In the subsequent ten years, lysosome-targeted MDVs have been associated with mitochondrial dynamics (especially fission events) [69], mitochondrial biogenesis [137], and MQC [138]. König et al. [136] demonstrated that lysosome-targeted MDVs were an alternative route to mitophagy, aimed at packing up and disposing mildly damaged mitochondrial components to lysosomes. Towers et al. also reported that MDVs compensated for the loss of LC3-mediated mitophagy in ATG7 knockout cells [139]. This mechanism allows cells to maintain the mitochondrial health through partial degradation, rescuing mitochondrial function before triggering global mitophagy or even apoptosis.

### Peroxisomes and mitochondrial dynamics

Peroxisomes are dynamic organelles that participate in lipid metabolism and ROS production [140]. Peroxisomes maintain intracellular lipid homeostasis by interacting with other organelles including the ER, mitochondria, and lysosomes, as well as lipid droplets [141]. Mitochondria are associated with peroxisomes both spatially and functionally. Peroxisomes are also targets of MDVs, contributing to mitochondrial turnover and quality control [142, 143]. These so-called "peroxisome-mitochondria contact sites" provide the very structural basis for their multiple functional synergy [144, 145], such as metabolic collaboration of mitochondria and peroxisomes (e.g., beta-oxidation of fatty acids and glyoxylate detoxification) [146], peroxisome-mitochondria redox interaction [147], and antiviral responses [148]. In addition, mitochondria can also share components of the mitochondrial fission machinery with peroxisomes to coordinate their own biogenesis [149, 150]. Under certain conditions, mitochondria (and possibly the ER) also contribute to peroxisome biogenesis (*de novo* formation) [151]. In addition, mitochondria show defects in several peroxisomal diseases, such as Zellweger syndrome [152], emphasizing the importance of the physiological interaction between the two organelles.

## Mitochondria at the Crossroad of Biomechanics and Bioenergetics

Based on the structural and functional interactions between the cytoskeleton and mitochondria, it would be appropriate to propose that mitochondria are mechanosensitive. In recent years, several studies have focused on mitochondria and mechanotransduction. For example, one study using atomic force microscopy showed that external pressure applied to cells promotes mitochondrial fission [153]. This force-dependent mitochondrial network disruption may represent a way to avoid mitochondrial damage when cells encounter deformation.

### Morphological evidence of mitochondria mechanosensing under force-loading

With the development of live-cell imaging and super-resolution microscopy, one can now observe the simultaneous reaction of mitochondria and cytoskeletal networks to external mechanical stress, providing evidence of mitochondrial mechanosensation. An *in vitro* study showed that fluid flow-induced primary cilia deflection promoted fatty acid oxidation and mitochondrial biogenesis in renal epithelial cells, increasing ATP production [154]. In another *in vitro* sustained stretch model of aberrant mechanical stimuli, apoptosis of cardiomyocytes was promoted by the release of cytochrome c from mitochondria [155]. Together, the mitochondrial membrane potential declined, which may have led to mitochondrial fragmentation. In a study of respiratory muscle weakness in a piglet mechanical ventilation model caused by impaired diaphragm contractility, the number of mitochondria was maintained, but the activity of complex IV of the ETC significantly decreased [156, 157]. This study demonstrated that mechanical ventilation eliminated the variability in tidal respiration compared to that of natural respiration; therefore, long-term abnormal and monotonous mechanical stimulation could lead to molecular changes in the mitochondria.

Cyclic mechanical strain enhances ROS production in endothelial cells [158]. Similarly, *in vitro* studies have shown that cyclic stretching induces the upregulation of ROS production in lung epithelial cells in a duration- and amplitude-dependent manner, which suggests that excessive expansion of the lung during mechanical ventilation might result in mitochondrial ROS-related lung injuries [159]. This study also provided evidence that global equibiaxial strain leads to partial mitochondrial stretching. When lung fibroblasts were placed under transient equibiaxial stretches of over 30%, acute mitochondrial rupture at discrete locations was observed immediately after stretching [160]. Researchers have used tetramethyl rhodamine methyl ester, a mitochondria-specific dye that senses the IM potential [161] and hence evaluates ATP production [162], to label and visualize mitochondria. Results of these studies indicated that external mechanical stimuli directly initiated fission events. Together, these studies prove that external mechanical stress, either transient or long-term monotonous, is capable of affecting the morphology and function of mitochondria.

As mentioned above, physiological mechanical stress on tissues and cells fluctuates. For example, beat-to-beat blood pressure shows significant variability and is elevated in hypertension [163]. In addition, breathing produces breath-to-breath variability in tidal volume [164]. The cellular signaling response toward mechanical stress is regulated by the magnitude and duration of the stress. Therefore, fluctuations in mechanical stimulation are likely to influence mechanotransduction. This unique mechanism is called fluctuation-driven mechano-transduction (FDM) [165]. It is reasonable that FDM was incorporated into all mechanosensitive cellular processes during evolution. While mechanotransduction has been investigated using static or cyclic stress, owing to limited laboratory techniques and conditions, these studies have always only employed monotonous stretch (MS). Fluctuations in cycle-by-cycle stretch or shear stress, called variable stretch (VS) or variable shear stress, can alter cellular behavior, such as bioenergetics and cytoskeletal organization [166, 167]. One study reported that FDM had a direct influence on mitochondria. Specifically, the ATP production rate in vascular smooth muscle cells (VSMCs) cultured under VS conditions was twice that in VSMCs cultured under MS conditions [166]. Moreover, VS affected the phosphorylation of ATP synthase and upregulated the expression of cytochrome c oxidase and its phosphorylated form. Interestingly, the expression of MFN1/2, but not DRP1, was promoted. VS also promoted mitochondrial biogenesis by increasing the key modulator of biogenesis, peroxisome proliferator-activated receptor gamma coactivator 1-alpha (PGC-1α), compared to that of MS [166, 168]. These biochemical changes are usually accompanied by various structural alterations, including elevation of the fractal dimension and coefficient of variation of MFs, MTs, and the mitochondrial network, suggesting that these structures are remodeled at high frequencies [166]. Investigations aimed at revealing the molecular mechanism of FDM showed that either ATP production or mitochondrial cluster size reduced in both groups, whereas VS could maintain a higher MMP than could MS. However, treatment with MT or vimentin assembly inhibitors eliminated the MMP difference. Inhibition of NMMII, DRP1, or mitotic kinesin-like protein 2 diminished MMP levels in VS cells compared to MS cells. Consequently, FDM-induced ATP production increased the level of myosin light chain phosphorylation in cultured VSMCs and *in vivo* aortic rings, which in turn generated a stronger contractile force in the aortic rings during VS [169]. In other words, VSMCs draw support from fluctuations in their mechanical milieu to induce reorganization and interaction of the cytoskeletal and mitochondrial networks, thereby leading to the accumulation of chemical energy stored in ATP. Comparing the phenotypes of unstretched, MS, and VS cells, the complexity of the cytoskeletal and mitochondrial networks developed in turn, accompanied by facilitated ATP production. Briefly, the results suggest that cells can harness energy from environmental fluctuations to charge the mitochondria. This is convincing evidence of fluctuations from the mechanical macroenvironment inducing molecular changes in the microenvironment. Additionally, this suggests that mechanical stimuli can directly act on the cytoskeletal and mitochondrial networks, thereby regulating oxidative phosphorylation and enhancing energy metabolism.

### Piezo ion channels link mechanotransduction to mitochondria

In 2010, Coste et al. [170] revealed a novel family of mechanically activated (MA) cation channels in eukaryotes, consisting of Piezo1/2 channels. Piezo1 channels exist within non-sensory tissues, mainly expressed in the lung, bladder, and skin, whereas Piezo2 channels predominantly exist in sensory tissues, such as sensory neurons of the dorsal root ganglion and Merkel cells [171]. Later, the molecular structure of the Piezo channel was partially revealed using low-temperature electron microscopy [172]. The Piezo channel is now recognized as a key player in multiple physiological and pathological processes, including volume regulation of erythrocytes [173], cell division and differentiation [174, 175], and innate immunity [176]. Mutations in Piezo1/2 are associated with various hereditary diseases, such as congenital lymphatic dysplasia [177] and muscular atrophy syndrome with perinatal respiratory distress [178].

Although direct evidence of Piezo channels regulating mitochondrial dynamics under mechanical stress has not yet been found, some indirect evidence supports this concept. Piezo1 channels are sensors of internal-plane tension, which allows transient entry of calcium to trigger numerous cell responses toward mechanical stimuli. Hence, it is suspected that mitochondria, known as the active and dynamic intracellular calcium pool like the ER, might respond to calcium transients caused by channel activation. Ellefsen et al. [179] reported the myosin 2 (Myo2)-based traction-assisted Piezo1 opening. In short, once stimulated by the tension stress generated in the adjacent cell membrane, focal adhesions subjected to Myo2-dependent strain opened the nearby Piezo1 channels, leading to consequent calcium influx. Interestingly, Crosas-Molist et al. (available at SSRN 3845005, 2021) reported that Myo2-dependent cell migration was regulated via AMPK and mitochondrial dynamics, suggesting a meshwork involving Piezo channels, Myo2, and mitochondria.

### AMPK connects mechanotransduction with mitochondrial dynamics

To survive the changing environment and unstable energy supply, eukaryotes have evolved a subtle system that regulates their metabolism according to their nutrient state. The core component of this system is AMPK, a heterotrimeric complex consisting of a catalytic alpha subunit along with regulatory beta and gamma subunits [180]. Under low-energy conditions, AMPK phosphorylates metabolism-specific enzymes to facilitate ATP production and attenuate ATP consumption [181]. Recently, the discovery of novel AMPK substrates has provided insight into the processes necessary for reprograming anabolism to catabolism [182, 183]. This energy switch controls multiple physiological processes, including cell growth, nutrient metabolism, and mechanotransduction.

There are several investigations reporting that AMPK responded to mechanical stimuli, which are related to calcium influx during mechanic stress but not in a calcium-dependent manner [184, 185]. Unfortunately, these studies lacked insights into the exact AMPK signaling reacting to mechanical stimuli. As mentioned above, mechanotransduction requires collaboration of various components, such as ECM, cytoskeleton, and so on. The interaction between these structures and AMPK is a strong hint of AMPK mediating mechanotransduction. Firstly, AMPK was found to modulate ECM remodeling in multiple tissues [186-188], indicating that AMPK itself could adjust extracellular mechanic environment. Recently, subcellular AMPK was shown to differentially react to fluid-flow via interplay with FAK and Src [28]. Guo et al. also reported similar results in breast cancer cells where AMPK in mitochondria were activated by intestinal fluid flow, and such reactions were blocked by FAK/Src inhibition [189]. Additionally, there is evidence that AMPK not only rapidly responds to external mechanics, but also initiates the assembly and movement of the cytoskeleton. Zhao et al. demonstrated that knockout of myosin-18B, a mediator of the assembly of Myo2 stacks, compromised phosphorylation of AMPK and myosin light chains, eventually resulting in the defects of cell migration [190]. Another study reported that AMPK suppression induced by the contractile inhibitor promoted the accumulation of MTs at the intercalated disk of cardiomyocytes, leading to enhanced cardiac contraction [191]. In brief, AMPK may serve as a master mechano-metabolic sensor in a variety of cells.

Further, AMPK has specific regulatory effects on mitochondrial homeostasis, such as sustaining the number of mitochondria by stimulating mitochondrial biogenesis, modulating mitochondrial dynamics, and regulating the surveillance of MQC via mitophagy [192, 193]. Mitochondrial depolarization and inhibition of mitochondrial ATP synthesis can interfere with energy metabolism, leading to an increase in the mitochondrial fission rate and/or a reduction in the mitochondrial fusion rate, which ultimately triggers mitochondrial fragmentation [194]. However, it is not completely understood how mitochondrial inhibitors (especially those that do not affect MMP) influence the morphology of the mitochondrial networks. Notably, agonists of mitochondrial fragmentation, such as ETC inhibitors, are promising activators of AMPK. Recent work showed that AMPK was indispensable in rotenone- and antimycin A- (inhibitors of ETC complex I and complex III, respectively) induced mitochondrial fission [195]. Direct activation of AMPK by small-molecule activators can also induce mitochondrial fragmentation [195]. In addition, two phosphorylation sites, Ser155 and Ser173, were unexpectedly found in MFF when identifying potential substrates for AMPK using proteomic and bioinformatic screening [195, 196]. Specifically, AMPK activation causes recruitment of DRP1 to the mitochondria, which is possibly dependent on phosphorylation of MFF Ser155 and Ser173 by AMPK [195]. It is speculated that the phosphorylation of MFF by AMPK could be a potential mechanism that explains the need for AMPK to promote mitochondrial fragmentation after ETC inhibition, and why a single activation of AMPK is sufficient to induce mitochondrial fission.

**Figure 4.**
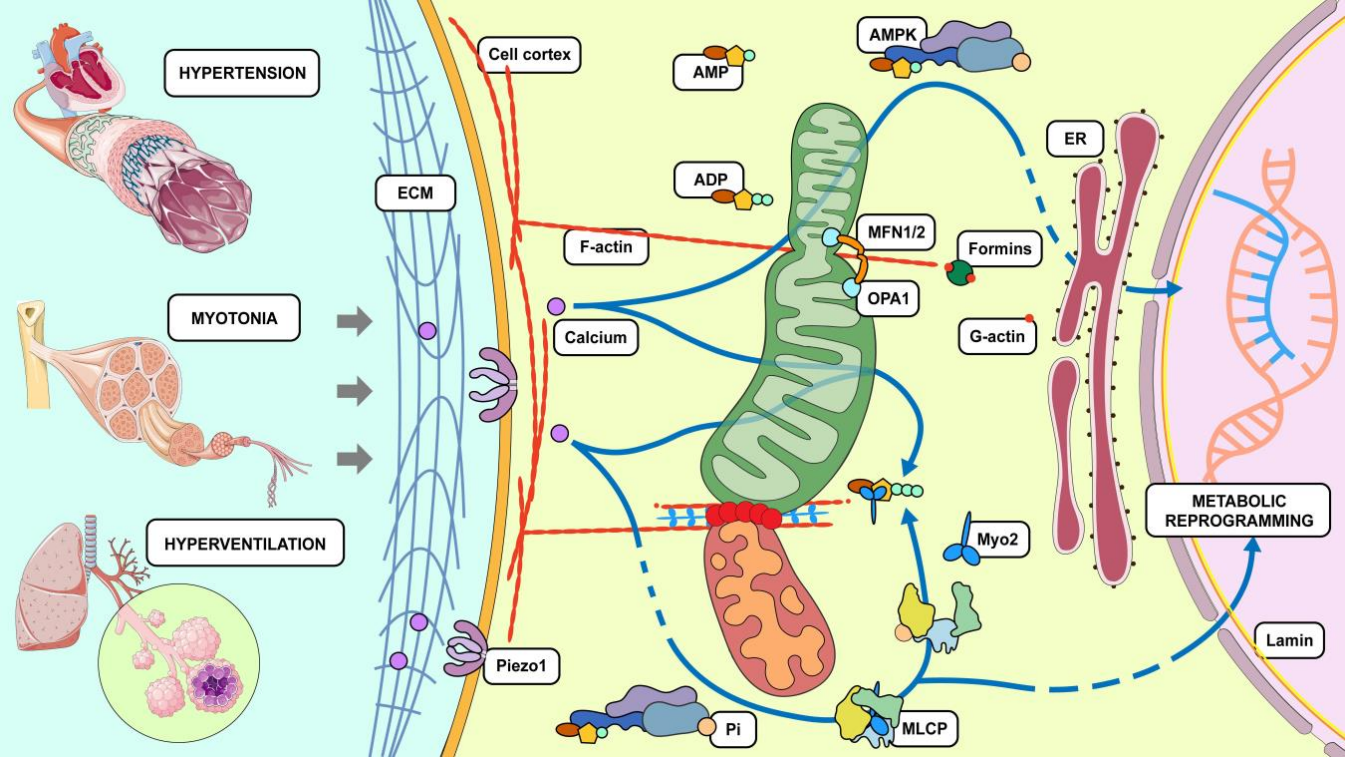
Mitochondria link mechanotransduction to metabolic reprogramming. Alterations in mechanical state result in pathological changes. Briefly, unfavorable mechanical stress is transduced into the cell through ECM deformation via the activation of mechanical sensors such as Piezos and morphological changes in the cytoskeletal system. Both mechanotransduction pathways involve dynamic changes in mitochondria and, therefore, alterations in energy production, which are likely regulated by AMPK signaling. Consequently, mechanical signals are transduced into the nucleus, leading to metabolic reprogramming, and resetting of cellular energy levels.

Here, we have annotated one creative opinion on the mechanism of AMPK linking mechanotransduction to mitochondrial dynamics. Recently, Crosas-Molist et al. (available at SSRN 3845005, 2021) reported that a weaker mechanical environment requiring less energy consumption led to fragmented mitochondria and decreased ATP production, which activated AMPK to promote cell mobility by enhancing Myo2 activity. Moreover, reducing adhesion, inhibiting mitochondrial fusion, and activating AMPK promoted cytoskeletal remodeling. These results suggested that AMPK may serve as the upstream sensor of mechanical stress instead of a downstream effector. AMPK can possibly regulate mechanic stimuli-induced mitochondrial dynamics through interaction with components of the mechanotransduction (Fig. 4).

## Mitochondrial dynamics, NLRP3, and inflammaging

Aging-related diseases have drawn increasing attention as population aging has become a worldwide concern. Aging conditions caused by inflammation, namely inflammaging, is believed to be the initiator and therapeutic target for multiple aging-related diseases [197]. NOD-like receptor family pyrin domain containing 3 (NLRP3) is an intracellular protein that is activated by harmful stimuli such as damage-associated or pathogen-associated molecular patterns [198, 199]. Activated NLRP3, Apoptosis-associated speck-like protein containing a CARD (ASC), and procaspase-1 assembled as one multiprotein complex, the NLRP3 inflammasome, which plays a vital role in inflammation. Abnormal NLRP3 activation and mitochondrial dynamics are linked to typical inflammatory diseases including neurodegeneration [197, 200].

Mitochondrial dynamics are crucial for maturation of the NLRP3 inflammasome during the activation of bone marrow-derived macrophage (BMDM) [201]. Kwon et al. [202] reported that lipopolysaccharide/ATP-induced assembly and activation of the NLRP3 inflammasome in BMDMs were enhanced by DRP1 inhibition, followed by fusion events. The fusion protein MFN2 promotes the connection between NLRP3 and mitochondrial antiviral-signaling protein (MAVS), enhancing the mitochondrial localization of NLRP3 and inflammasome activation [203]. MAVS also increases the association between NLRP3 and MFN2. In addition, such an association depends on MMP [204]. Physiological MMP is necessary for interactions among NLRP3, MFN2, and MAVS during mitochondrial fusion, which subsequently leading to the activation of the NLRP3 inflammasome [205]. Aberrant MMP or MFN2 knockdown attenuates NLRP3 inflammasome activation, resulting in lower IL-1β production.

Wang et al. [206] reported controversial results that receptor-interacting protein kinase (RIP)1-RIP3-DRP1 signaling was involved in viral infection-induced activation of the NLRP3 inflammasome. In BMDMs, several RNA viruses, such as *Indiana vesiculovirus*, enhance the binding of RIP1-RIP3. The bound RIP1-RIP3 complex can then phosphorylate DRP1 at S616, which helps in the DRP1 translocation to the mitochondria where it catalyzes mitochondrial fission and arrests metabolism, activating the NLRP3 inflammasome. In addition, interrupted fission events can recruit NLRP3 to the mitochondria, promoting NLRP3 inflammasome assembly and activation [201].

DRP1 plays an important role in early cerebral injury [207]. DRP1 mediated mitochondrial fission activates astrocytes and induces inflammation by releasing fragmented mitochondria into the surrounding environment. Therefore, DRP1 is suggested to be a promising target for regulating neural inflammatory diseases originating from dysfunctional mitochondrial dynamics. In a related experiment, treatment with a selective DRP1 inhibitor, Mdivi-1, attenuated mitochondrial damage and ROS produced by active fission events, thereby reducing activation of the NLRP3 inflammasome [208]. Moreover, ethanol-induced calcium overload, a vital mechanism in neurodegeneration, can promote calcium influx, resulting in calmodulin-dependent protein kinase II activation, leading to DRP1 S616 phosphorylation [209]. Phosphorylated DRP1 translocates to the mitochondria, intervenes in mitochondrial dynamics, and promotes NLRP3 inflammasome activation, inducing chronic neural inflammation [210]. Further investigations are required to reveal additional interesting features of the regulatory mechanism of mitochondrial dynamics in NLRP3 and inflammatory diseases.

## Summary and Perspectives

Mitochondria provide a stable energy supply for various cellular activities, including cytoskeletal dynamics and ECM remodeling. Mitochondrial dynamics are an important approach to modulating mechanotransduction at the metabolic level. Mitochondria are directly connected to several components of the cytoskeleton; therefore, mechanical stress can be transmitted to the mitochondria independent of signal transduction molecules, such as calcium. Such mechanical force transmission can lead to direct morphological changes in the mitochondria, e.g., fragmentation and elongation. Meanwhile, deformation of cytoskeletal filaments due to mechanical stress may modulate mitochondrial dynamics, similar to the role of the cytoskeletal system in regulating mitochondrial functions.

A dozen interactive events occurring between mitochondria and other intracellular membranous structures have recently been uncovered. The ER is first on the shortlist of mitochondria-associated organelles, followed by the nucleus and lysosomes. In these studies, mitochondria manifested dynamic alterations in response to mechanical stress by establishing contact sites with the membranes of other organelles and were dynamically regulated. It should be noted that mitochondria do not always require direct connections to complete their dynamic activities. For instance, MDVs are representative patterns of mitochondrial dynamics that promote mitochondrial turnover and quality control. Owing to advancements in technology, such as live-cell imaging and cryo-electron microscopy, many novel vesicle-based interactions, characterized as covert but frequent communication between mitochondria and other organelles, have been uncovered. Currently, such “whispers” are already present in the mitochondria, ER, and Golgi apparatus [211]. Considering that vesicles are ubiquitous vehicles for interorganelle crosstalk, it is predictable that mitochondria also interplay with more membranous cellular components via specifically labelled vesicles that carry through mechanotransduction among intracellular compartments.

Finally, mitochondria, the cytoskeletal system, and membranous organelles are presumed to be functional complexes that mediate mechanical stress sensation and corresponding metabolic alterations. Mitochondrial dynamics as a whole are therefore the nexus linking the three parts, serving as the crucial mechanism modulating the force-to-metabolism process. Hence, mitochondrial dynamics could be a promising drug target for many diseases, especially those characterized as constantly experiencing variable mechanical environments or irregular metabolic coping toward normal mechanical stress. These new findings and techniques will ensure a new era in cellular physiology and precision medicine.
